# The prediction of virus mutation using neural networks and rough set techniques

**DOI:** 10.1186/s13637-016-0042-0

**Published:** 2016-05-13

**Authors:** Mostafa A. Salama, Aboul Ella Hassanien, Ahmad Mostafa

**Affiliations:** 1 British University in Egypt (BUE), Cairo, Egypt; 2 Cairo University, Cairo, Egypt; 3 Scientific Research Group in Egypt, (SRGE), Cairo, Egypt

**Keywords:** Gene prediction, RNA, Machine learning

## Abstract

Viral evolution remains to be a main obstacle in the effectiveness of antiviral treatments. The ability to predict this evolution will help in the early detection of drug-resistant strains and will potentially facilitate the design of more efficient antiviral treatments. Various tools has been utilized in genome studies to achieve this goal. One of these tools is machine learning, which facilitates the study of structure-activity relationships, secondary and tertiary structure evolution prediction, and sequence error correction. This work proposes a novel machine learning technique for the prediction of the possible point mutations that appear on alignments of primary RNA sequence structure. It predicts the genotype of each nucleotide in the RNA sequence, and proves that a nucleotide in an RNA sequence changes based on the other nucleotides in the sequence. Neural networks technique is utilized in order to predict new strains, then a rough set theory based algorithm is introduced to extract these point mutation patterns. This algorithm is applied on a number of aligned RNA isolates time-series species of the Newcastle virus. Two different data sets from two sources are used in the validation of these techniques. The results show that the accuracy of this technique in predicting the nucleotides in the new generation is as high as 75 %. The mutation rules are visualized for the analysis of the correlation between different nucleotides in the same RNA sequence.

## Introduction

The deoxyribonucleic acid (DNA) strands are composed of units of nucleotides. Each nucleotide is composed of a nitrogen-containing nucleobase, which is either guanine (G), adenine (A), thymine (T), or cytosine (C). Most DNA molecules consist of two strands coiled around each other forming a double helix. These DNA strands are used as a template to create the ribonucleic acid (RNA) in a process known as transcription. However, unlike DNA, RNA is often found as a single-strand. One type of RNA is the messenger RNA (mRNA) which carries information from the ribosome, which are where the protein is synthesized. The sequence of mRNA is what specifies the sequence of amino acids the formed protein. DNA and RNA are also the main component of viruses. Some of the viruses are DNA-based, while others are RNA-based such as Newcastle, HIV, and flu [1]. RNA viruses are different than the DNA-based viruses in the sense that they have higher mutation rates, and hence, they have higher adaptive capacity. This mutation causes a continuous evolution that leads to host immunity, and hence, the virus becomes even more virulent [2]. One of the RNA virus mutations is the point mutation which is a small scale mutations that affects the RNA sequence in only one or few nucleotides, such as nucleotide substitution. This substitution refers to the replacement of one nucleobase (i.e., base) either through transition or transversion. This substitution refers to the replacement of one nucleobase (i.e., base) by another either through transition or transversion. Transition is the exchange of two purines (A <−> G) or one pyrimidine (C <−> T), while transversion is the exchange between a purine and pyrimidine [3, 4]. Another type of point mutation is frame-shift which refers to the insertion or deletion of a nucleotide in the RNA sequence.

One of the important focuses in the field of human disease genetics is the prediction of genetic mutation [5]. Having information about the current virus generations and their past evolution could provide a general understanding of the dynamics of virus evolution and the prediction of future viruses and diseases [6]. The evolutionary relationship between species is determined by phylogenetic analysis; additionally, it infers the ancestor sequence of these species. These phylogenetic relationships among RNA sequences can help in predicting which sequence might have an equivalent function [7]. The analysis of the mutation data is very important, and one of the tools used for this purpose is machine learning. Machine learning techniques help predict the effects of non-synonymous single nucleotide polymorphisms on protein stability, function and drug resistance [8]. Some of these techniques that are used in prediction are support vector machine, neural networks and decision trees. These techniques have been utilized to learn the rules describing mutations that affect protein behavior, and use them to infer new relevant mutations that will be resistant to certain drugs [9]. Another use is to predict the potential secondary structure formation based on primary structure sequences [10–12]. A different direction is to predict the discovery of single nucleotide variants in RNA sequence. Another tool in machine learning is Markov chains, which can describe the relative rates of different nucleotide changes in the RNA sequence [13]. These models consider the RNA sequence to be a string of four discrete states, and hence, tracks the nucleotide replacements during the evolution of the sequence. In these models, it was assumed that the different nucleotides in the sequence evolved independently and identically, and justified that using the case of neutral evolution of nucleotides. Following that, several researches developed methods negated that assumption and identified the relevant neighbor-dependent substitution processes [14].

The prediction of the mutated RNA gives a clear understanding of the mutation process, the future activity of the RNA, and the help direct the development of the drugs that should be designed for it [15, 16]. In this work, we propose a machine learning technique that is based on rough set theory. This technique predicts the potential nucleotide substitutions that may occur in primary RNA sequences. In this technique, a training phase is utilized in which each iteration the input is an RNA sequence of one generation of the virus, while the output is the RNA sequence of the next generation of the virus. Every feature in the input is a nucleotide in the RNA sequence corresponds to a feature in the output. The training of the machine learning technique is fed with aligned RNA sequences of successive generations of the same RNA viruses that exhibited similar environmental conditions. The technique introduced in this paper predicts the last RNA sequence based on the previous RNA generation sequence. Following that, the predicted RNA sequence is compared the actual RNA sequence in order to validate the ability of the machine learning technique to predict the RNA evolution and this prediction accuracy. This comparison results in a percentage which is calculated based on the number of matched nucleotides between both the predicted and actual RNA sequence versus the total number of nucleotides in the sequence. One of the main important methods of this technique is that it extracts the rules governing the past mutations, and hence, is able to infer the possible future mutations. These rules show the effect of a set of nucleotides on the mutation of their neighboring nucleotide. This technique visualizes these rules to allow an integrated analysis of the mutations occurring in successive generations of the RNA virus. Besides using the Rough set theory in our technique, a traditional machine learning technique is utilized which is neural networks in order to clarify the prediction process and validate our results. Finally, we present a way to reform the RNA alignments of a set of successive generations of the same virus. This reformation step is important in order to fit the input requirements for any machine learning technique.

This paper presents a proof of concept by applying this technique on a set of successive generations of the Newcastle Disease Virus (NDV) from two different countries, Korea and China [17, 18]. In these sets, the percentage of nucleotides that exhibits variation from a generation to another is 57–65 % for the two used sets of sequences. The proposed techniques percentage accuracy in the prediction of the varied nucleotides in the tested sequence in the last generation are 68–76 %. Although these results are not statistically significant at this point, it still however proves the applicability of the proposed technique. It is worth noting that the learning and accuracy of prediction of this technique increases as the number of instances in the data set increases. The rest of the paper is organized as follows. Section 2 presents the related work in applying machine learning techniques in genetic problems, as well as the proposed technique to solve these problems. The experimental work and discussion appear in Sections 3 and 4, and finally, the conclusion is presented in Section 5.

## Methods

### Related work

Predicting techniques have been utilized in the field of genetics for many years, and have been geared towards different directions. One of these directions is the detection of the resistance of the virus to drugs after its mutation [19], in which, machine learning techniques are focused on learning the rules characterizing the aligned sequences that are resistant to drugs. The rules will be later on used to detect the drug resistance gene sequences amongst a set of testing sequences. The training phase of these techniques works by having each protein sequence represented as a feature vector and fed as the input to this technique [20]. An example of these machine learning techniques are support vector machine (SVM) and neural networks, which can be trained on data sets of drug-resistant and non-resistant DNA sequences of virus population. In the training phase, these techniques learn the rules of classifying new generations of the virus a being drug-resistant or not [21]. However, these techniques are black box techniques, that cannot be used to infer any information about the rules used in this classification. Another disadvantage is that these techniques utilize 20 bit binary numbers instead of characters as a representation for 20 different amino acids condons. This increases the size of the input to the used algorithm, which in return increases the complexity of the classification process. However, the authors in [22] introduced a technique that uses characters instead of number as the input, and they utilize a decision tree in order to provide direct evidence on the drug resistance genes through a set of rules. Their technique trains and tests its efficiency on 471 isolates against 14 antiviral drugs, creating a decision tree for each drug. The input of this decision tree is the isolates sequence in which, each position is one of 20 naturally occurring amino acids represented in characters. The results of this technique concludes whether the virus is a drug-resgstenc virus or not. An example of this generated decision tree is: if the codon at position 184 is for methionine (M) and the codon at position 75 for alanine (A), glutamic acid (E) or threonine (T), then the virus carrying this gene is resistant to (3TC) antivirus drug. Another example is the detection of whether a point mutation in a cell is transmitted to the offspring or not, i.e., gremlin mutation vs. somatic mutation [23].

Another research direction is the prediction of the secondary structure of the RNA/DNA of the organism in the generation post-mutation. The ribonucleic acid (RNA) molecule consists of a sequence of ribo-nucleotides that determines the amino acids’ sequence in the protein. The primary structure of the RNA molecule is the linear form of the nucleotides’ sequence. The nucleotides can be paired based on specific rules that is: adenine (A) pairs with uracil (U) and cytosine (C) with guanine (G). Base pairs can occur within single-stranded nucleic acids. The RNA sequence is folded into secondary structure in which a pair of basis is bonded together. This structure contains a set of canonical base pairs, whose variation is considered as a form of mutation that can be predicted. Several researches have been focused on automating the RNA sequence folding [24] and predicting the secondary structure form [25]. The probability of the generation of any secondary structure is inversely proportional to the energy of this structure. This energy is modeled based on extensive thermodynamic measurements [26]. Applications like “RNAMute” analyzes the effects of point mutations on RNAs secondary structure based on thermodynamic parameters [27].

The third research direction is the prediction of single nucleotide variants (SNV) at each locus, i.e., nucleotide location. The SNV existence are identified from the results of the Next Generation Sequencing (NGS) methods [6]. NSG is capable of typing SNP, which is the mutation that produces a single base pair change in the DNA sequence. For example, the two sequenced DNA fragments from different generations are AAGCCTA and AAGCTTA. Noticed that the fifth single nucleotide C in the first fragment varied to a T in the second fragment. This genotype variation change represents the mutation in the child genomes of the next generation. The steps of allocating the SNVs start with collecting a set of aligned sequences from NGS readings analyzed against a reference sequence. At each position *i* in the genome data, the number of reads *a*_*i*_ that match the reference genome and the number of reads *b*_*i*_ that do not match the reference genome are counted. The total number of reads, depth, is given by *N*_*i*_=*a*_*i*_ + *b*_*i*_. A naïve approach to detect the SNV locations is to find the location *i* ∈ [1, T] whose fraction *f*_*i*_=*a*_*i*_ / *N*_*i*_ is less than a certain threshold [28].

Although this approach is accurate for large number of generations, usually this is not case due to low collected number of sequences. Moreover, it only predicts the existence of the SNV, however, it does not predict the future sequence. A model is proposed in [29] to infer the genotype at each location. The model characterizes each column in the alignment to be one of three states, the first Homozygous type is where all genotypes are the same as the reference allele states, while the second types is where all genotypes are the same as non-reference allele, the last type is for mixture of reference and non-reference alleles. This model calculates posterior probability P(*g* | *x*,*z*) of the genotype at position *u* in the current sequence *z*, given a reference sequence *x* and the sequence z. The genotype highest posterior probability (MAP) is selected. The detection of the third state is based on SNVMix model [30], which uses the Bayesian Theorem and MAP to calculate the posterior probabilities for a mixed genotype *g*_*m*_. In this case, the *P*(*g*_*m*_|*a*_*i*_) can be calculated as shown in Eq. 1 where *a*_*i*_ is the number of reads that matches the reference at location *i*.

1 $$ P(g_{m} | a_{i}) = P(a_{i} | g_{m}) * P(g)  $$

The prior probability *P*(*a*_*i*_|*g*_*m*_) is calculated using the binomial distribution *dbinom* in the case of mixed genotype [30] as shown in Eqs. 2, 3, and 4. The parameter $\mu _{g_{m}}$ of the *dbinom* is of value 0.5 where the probability of the genotype, matching or not matching, is equal. And *N*_*i*_ represents the total number of generations.

2 $$ P(a_{i} | g_{m}) \approx dbinom(a_{i} | \mu_{g_{m}},N_{i})  $$

3 $$ P(a_{i} | g_{m}) = \binom{N_{i}}{a_{i}} \mu_{g_{m}}^{a_{i}} \left(1 - \mu_{g_{m}}^{N_{i} -_{i}}\right)  $$

4 $$ P(a_{i} | g_{i\{ab\}}) = \binom{N_{i}}{a_{i}} \frac{1}{2^{N_{i}}}  $$

These three research direction focus on predicting the activity of the newly evolved viruses. A different direction is the prediction of the rates of variation of the nucleotides in the RNA sequences of successive generations of the virus [31]. In this direction, models are introduced to analyze the historical patterns and variation mechanism of the virus and detect the rates of variation of each point substitution of its RNA sequence. These models are characterized as either neighbor-dependent or non-dependent variation.

The contribution in this paper focuses on the prediction of the RNA sequence of newly evolved virus by detecting the possible point mutations. The nucleotide substitutions of the coding regions in this Newcastle virus occurred frequently [32]. These substitutions and dependency between nucleotides is captured to form a set of rules that describes the evolution of the RNA virus.

### The proposed algorithm and discussion

The focus of this paper is to analyze the changes and mutations that occur after each generation of the virus. This analysis is done through monitoring these changes and detecting their patterns. The mutation of the RNA viruses are characterized by high drug-resistance, however, these mutations can be predicted by applying machine learning techniques in order to extract their rules and patterns. The proposed algorithm here is applied on a data set of aligned RNA sequences observed over a period of time. This data was collected and presented in previous research [17, 18], in which all animal procedures performed were reviewed, approved, and supervised by the Institutional Animal Care and Use Committee of Konkuk University. This space of potential virus mutations provides a proper data set required for the computational methods used in the algorithm. The preprocessing is started by the alignment of RNA sequence for the purpose of predicting the evolution of the virus based on the gathered sequences. The steps performed are the mining and learning the rules of the virus mutation, in order to predict the mutations to help creating new drugs. The input data set is a set of aligned DNA sequences. The RNA isolates are ordered from old to new according to the time of getting the virus and isolating the RNA. The machine learning techniques used in this step are neural networks, and a proposed technique that is based on the rough set theory. An important step in the proposed technique is building the decision matrix required for rule extraction, which is based on the idea that if the value of the attribute has no effect on describing the category of the object, then this attribute can be excluded from the rule set [33]. An important clarification is that every nucleotide in both the neural networks and proposed technique act as the target class label required in the classification. These target nucleotides will have one of four different genotype values that will be predicted by the classifier.

The preprocessing step includes the feature selection which is important in decreasing the processing cost by the removal unnecessary nucleotides. These nucleotides are those that do not change during the different generations of the virus, hence, they do not add any information in the classification/prediction step. This is due to the fact that not changing across RNA generations imply that they have no effect on the mutated nucleotides. Hence, their existence will have no effect, and will moreover deteriorate the net accuracy prediction result. Although this step is important as a preprocessing step in the neural network methods, it can be avoided in the proposed rough set based technique since the technique removes the non-required nucleotides internally.

#### Neural network technique

**Fig. 1:**
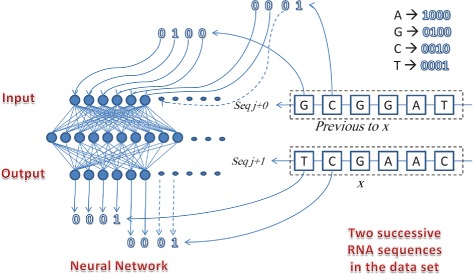
The learning of the neural network from the input data set

Neural networks technique can be used to predict different mutations in RNA sequences. The first step of this technique is to specify the structure of both the input and output. The number of nodes in the input and in the output of the neural networks is the four times the number of nucleotides in the RNA sequence. Hence, each nucleotide will be scaled to 4 binary bits, i.e., if the nucleotide is “A”, then the corresponding bit will be 1. However, it is not correct to transform the letters to numerals, for example the nucleotide values of [A, C, G, T] to [0, 1, 2, 3]. The reason being is that the distance between each nucleotide is the same, while the distance between [0–3] is not the same as that between [0–1]. The effect of this can be demonstrated by the following example: when applying neural networks to the numerically transformed sequence, the results show unsuccessful results. This is because the neural networks technique changes the values of the output based on the nucleotide values. Hence, the backward propagation in the case of moving from the value *T* to the value *A* will not be the same as moving from *T* to *G*. This is considered a mistake in the learning phase of the neural network, and it will lead to incorrect classification and prediction. The same case occurs when applying support vector machine and Bayesian belief networks. The training of the neural network will occur first by considering every scaled RNA segment s one training input to the neural network. The desired output corresponding to the input DNA segment is the next successive scaled RNA segment from the next generation of the training data set. As shown in Fig. 1, for every input/output sequence, the weights are continuously updated until accuracy exceeds 70 %. This accuracy is calculated according to the number of correct predicted nucleotides to the total number of nucleotides in the sequence. After the accuracy exceeds 70 %, the next input is the output DNA sequence in the current step. The last RNA segment is left for testing.

The disadvantage in using a neural network technique or support vector machine is the scaling of each nucleotide in the DNA sequence to four input states. This scaling process increase the computational complexity of the technique. On the other hand, the limited number of input instances could negatively affect the accuracy of the prediction process. Finally, the extraction of the rules in this technique is not possible, and hence, the prediction process is not feasible.



#### Rough set gene evolution (RSGE) proposed technique

This paper proposed a new algorithm for solving the evolution prediction problem that is based on the input time series data set. Each RNA sequence is mutated, i.e., evolution passage, to the next RNA sequence in the data set, as these sequences are sorted from old to recent dates. For each iteration in this learning phase of this algorithm, the input is an RNA sequence and the output is the next RNA sequence in the data set. The learned output is not the classification of the RNA virus; however, it is the RNA virus after mutation. Because, techniques like support vector machine, neural networks, and Bayesian belief networks have to deal with data in a numerical form, the proposed technique deals with alphabetical and numerical data in the same fashion.

The purpose of this technique is to infer the rules that governs certain value [*A*,*C*,*G* *o* *r* *T*] for each nucleotide. At this point, the training applied will consider each nucleotide as a target class of four values. The input that leads to one of the values of the target class is the sequence before the sequence containing the current value of the target class. The machine learning algorithm will learn what input will produce which output accordingly. The rules learned from the used machine learning algorithm will be used to predict the generated nucleotide corresponding to the input. The rule will be in the form of short sequences of nucleotides genotype and location that govern the mutation of a nucleotide from certain genotype to another. The number of iterations will be the number of nucleotides in the RNA sequence. After each iteration, the required rule for the nucleotide x corresponding to the current iteration will be extracted.

**Fig. 2:**
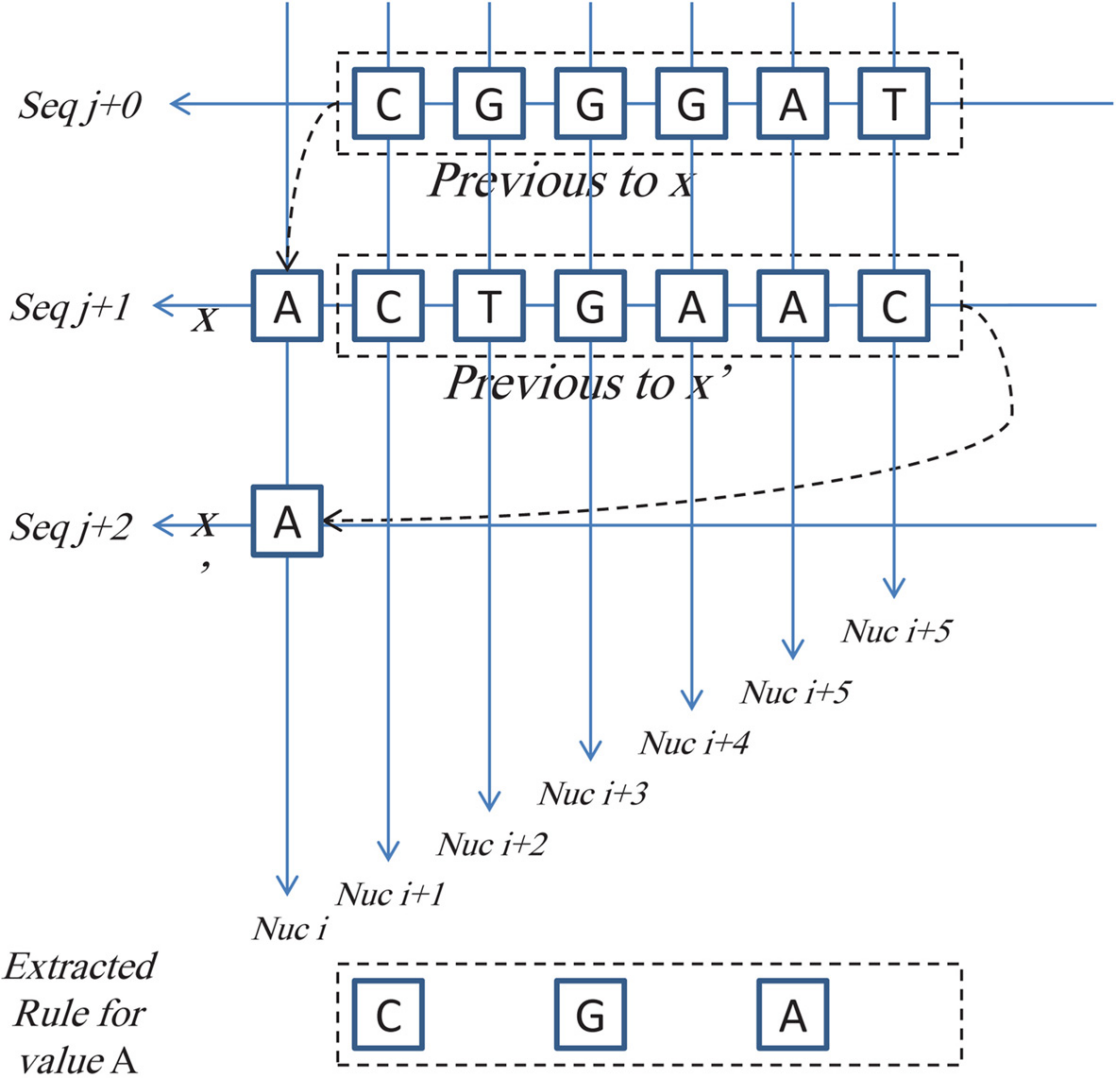
Nucleotide i for iteration i in the proposed algorithm, nucleotide as position i is the same, not changed

**Fig. 3:**
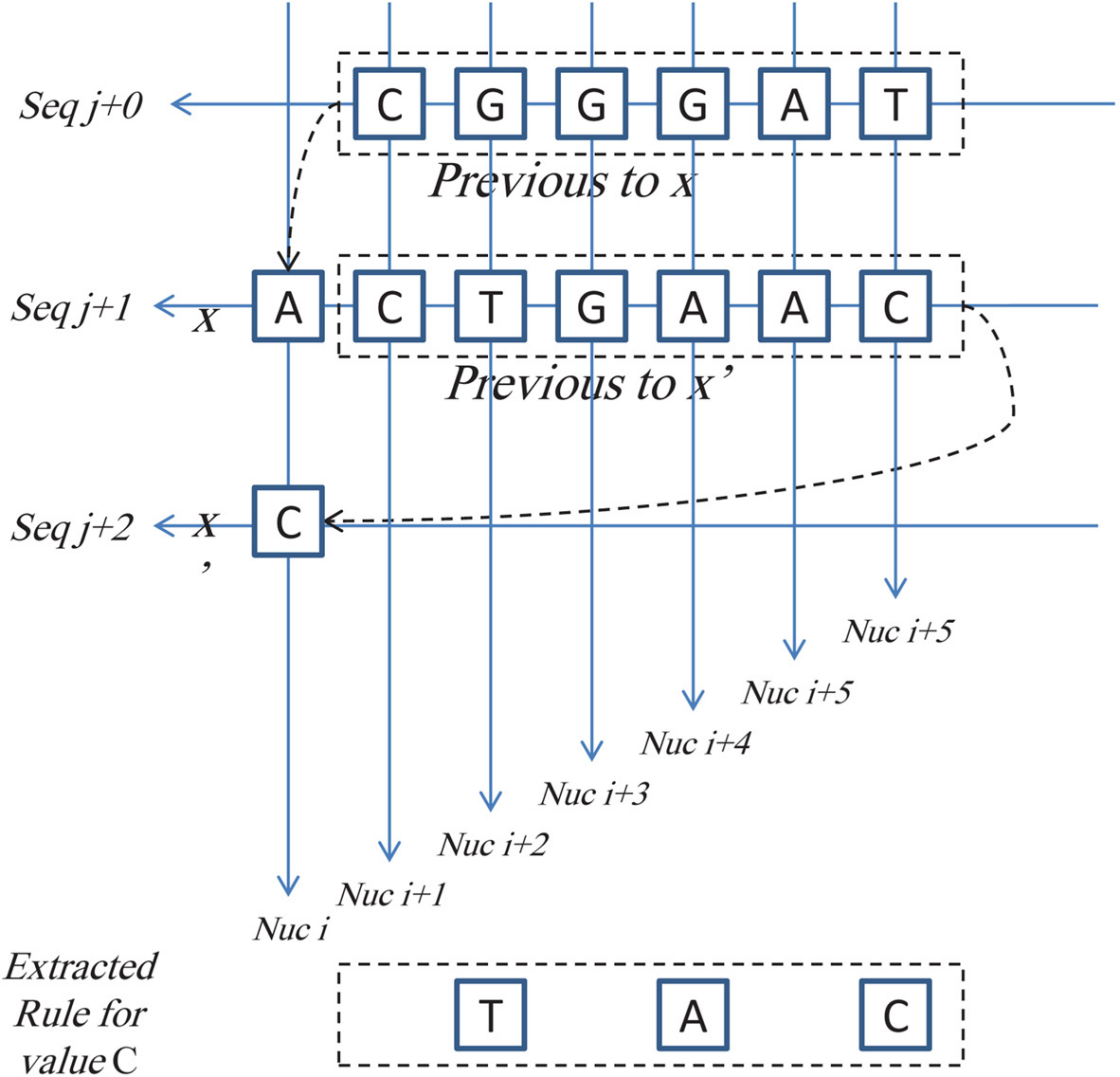
Nucleotide i for iteration i in the proposed algorithm, nucleotide as position i is the not the same, changed

In each iteration, the algorithm detects the value of nucleotide *x* in the sequence in the data set, as well as the value of this same nucleotide in the next sequence *x* ^′^. The following step in the algorithm is the detection of the values of all nucleotides in the preceding sequences to the ones that include nucleotides *x* and *x* ^′^. This step is applied to detect the sequence of the previous nucleotide values and leads to the value of the nucleotide under investigation. This is illustrated in Figs. 2 and 3. In Fig. 2, the sequence [*C* *G* *G* *G* *A* *T*] precedes nucleotide *x* at position *i* of value *A*, and the sequence [*C* *T* *G* *A* *A* *C*] precedes nucleotide *x* ^′^ at position *i* of value *A*. The nucleotide *x* in the position *i* corresponding to the current iteration does not change from sequence *j*+1 to sequence *j*+2. In this case, if the nucleotides in the sequence preceding to the one corresponding to the nucleotide *x* do not change, then these nucleotide values will be included in the rule of nucleotide *x*, otherwise they will be completely excluded. Hence, for Fig. 2, the extracted rule for the value *A* of the nucleotide at position *i* will be [*A*:*i*,*C*:*i*+1,*G*:*i*+3,*A*:*i*+5]. A different case is presented in Fig. 3, where the value of the nucleotide at position *i* is changed from value *A* to value *C*. In this case, if the nucleotides in the sequence preceding the one corresponding to the nucleotide *x* changes, then these nucleotide values will be included in the rule of nucleotide *x*, otherwise they will be completely excluded. Hence, for Fig. 3, the extracted rule for the value *C* of the nucleotide at position i will be [*A*:*i*,*T*:*i*+2,*A*:*i*+4,*C*:*i*+6].

The reason behind using this methodology in extracting the rule is that for each iteration in this algorithm is that two main cases are considered. The first case is that the value of the iterated nucleotide corresponding to this iteration does not change. In this case, if the neighbor nucleotides to this iterated nucleotide do not change, this will indicate that the values of the nucleotides are attached and leads to the value of the iterated nucleotide. If the neighbor nucleotides did change, then the variation of these values do not affect the iterated nucleotide, and hence they can simply be removed from the extracted rule. The second case is considered the opposite of the previous one where the existed nucleotides in the RNA sequence will cause the change of the genotype of a specific nucleotide in the following generation. In this case, the extracted rule of the genotype at this nucleotide location will include the set of neighbor nucleotides. While the unchanged behavior of some nucleotides means their unimportance or non-effect of changing the value of the of iterated nucleotide. As shown in Algorithm 1, if the number of sequences is *N*, and the number of nucleotides is *K*, the computational complexity of the calculations is as follows:

5 $$ Algorithm Complexity = O(K*N * (2*K)) = O(N * K^{2})  $$

## Results

The analysis of RNA mutations requires the gathering and preparing a set of aligned RNA sequences that go through different mutations over a long period of time. A set of time-series successive isolates of the Newcastle virus RNA are collected from two different countries, China and South Korea [17, 18]. The GenBank accession numbers of NDVs isolates recovered from live chicken markets in Korea in year 2000 are AY630409.1-AY630436.1, and from healthy domestic ducks on farms are EU547752.1-EU547764.1. The total number of isolates for this data set is 22, each isolate is of 200 nucleotide. While the accession numbers of sequenced isolates extracted from chicken in China in years from 2011 to 2012 are KJ184574-KJ184600 [34]. The total number of isolates for this data set is 45, each isolate is of 240 nucleotide. Each set of isolates is listed and sorted according to the date of extraction for time series analysis of the evolution of the NDV virus. The experimental is applied only a partial F gene sequence of NDV from the GenBank record. The virus RNA sequences were monitored and collected retrospectively at regular intervals from similar animal type. The intervals between successive RNA sequences in the Chinese dataset is short relative to the Korean data set. The difference between both types of intervals does not affect neither the analysis process nor the accuracy of the prediction. The extracted data examined for the Korean and Chinese datasets are represented by two different regions of viral genome with the different lengths (200 and 240). It is important to clarify that the two input data sets can not be merged because each input set of segments are aligned separately. So it is impossible here to apply prediction performance of the proposed models on the Chinese dataset using the Korean dataset for training.

**Fig. 4:**
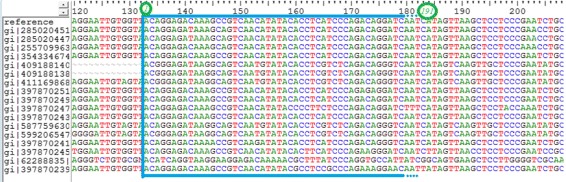
Aligned gene sequence of nucleotides

Figure 4 shows a segment of these aligned RNA sequence, in which, we select only the columns that contain missing values. It is clear in this figure that some nucleotides columns are passing through mutation along the time period under examination, while the other nucleotides do not witness any change during it. Applying any machine learning technique is composed of two main steps, the first is the training of the first part of the input data set, while the second step is using the rest of the data for testing. The classification accuracy percentage is the ration of correctly predicted nucleotide divided to the total number of nucleotides in the sequence.

The Chinese data set shows 33 % of the total number of nucleotides have been mutated, and this percentage in the Korean is 43 %. The rate of genome mutations over the time period applied shows variable number mutations.

### Neural network (NN) results

**Fig. 5:**
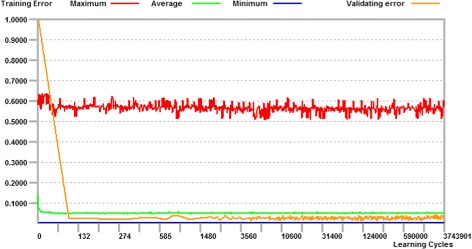
Neural network classification results

In order to apply NN to the aligned time series RNA sequences, only a part of the sequence will be considered. The partial training of the RNA sequence is applied to ensure a reasonable training execution time. Applying neural networks to this number of target class labels will lead to a very high processing complexity. The training phase contains only 20 nucleotides, where each nucleotide will be scaled to only four input nodes. This will lead to a data set of 80 input nodes and 80 output nodes. For the Korean input data set, the in-out for the training is the first 20 out of 22 instances. The target 20 output instances for these 20 input instances started from the second instance in the input data set and ends at the 21^*st*^ instance. The testing will have the 21^*st*^ RNA sequence as the input to the neural networks and the 22^*nd*^ RNA sequence as the target output. The results show that after 3743901 learning cycles of back-forth propagation, the validation accuracy reaches 70 % percent as shown in Fig. 5.

### Rough set gene evolution (RSGE) results

The validation of the proposed rough set gene evolution (RSGE) is achieved through testing the total number of nucleotides. For the Korean input data set where the total number of nucleotides is 200, the number of correct nucleotide matches is 148, which is considered 74 % classification accuracy percentage. Also, only four nucleotides have shown incorrect matches, which corresponds to 2 % out of all nucleotides. 48 nucleotides have shown no matching results, which indicates that none of the four rules of each of these nucleotides is applied. These results show the predicted and actual target RNA sequence of nucleotides.

For Korea Data Set Newcastle disease virus strain Kr-XX/YY [1982–2012]:

- Actual : ATGGGTTCCAAATCTTCTACCAG
- Predicted : ATGGGNNCCANANCTTCTACCAN

For the Chinese input data set where the total number of nucleotides is 240, the number of correct nucleotide matches is 180, which corresponds to 75 % classification accuracy. Also, only four nucleotides, i.e., 1.6 %, have shown incorrect matches. And, 56 nucleotides have shown no matching results, which indicates that non of the four rules of each of these nucleotides is applied. The results for the China Data Set Newcastle disease virus isolate JS XX XX Ch [2011–2012] is:

- Actual : ATGGGCTCCAAACCTTCTACCAG
- Predicted : TGGGCTCCAAACNTTCTACCNG

## Discussion

A sample of the generated rules is figured as follows:

- For Nucleotide P131:
  - If Genotype is A then P47=C, P57=A
  - If Genotype is G then P23=A
- For Nucleotide P101:
  - If Genotype is C then P6=T, P118=C
  - If Genotype is T then P6=G, P118=A

This sample is composed of two base nucleotides only in the RNA sequence, which are 131 and 101. The first rule shows that the nucleotide at position 131 will take the genotype value “A” in the following generation if the nucleotide at position 47 in the current generation is ’C’ and the one at position 57 is of type “A”. These rules show the existence of a correlation between the three nucleotides, i.e., those at positions [131, 47, and 57], in the first rule and [101, 6 and 118]. The genotype values of some specific nucleotides could affect the alteration/mutation of the RNA sequence. The input Chinese and Korean data sets contain different genotypes, AA, AB, and BB. The nucleotides of the genotypes that have the value AA and BB can be excluded from the resulted rule set. These genotypes need not to be predicted as the values are the same over all the generations. Table 1 shows the extracted rule sets of China countries for the AB nucleotides only. On the other hand, a similar table of rules is generated for the Korean data set.

**Table 1:** AB genotype rules for the Chinese data set

Nucleotide	Predicted	Rule	*N*_*sequence*_
position	genotype		
*N*_33_	T	*N*_37_=C	CCCCCCCCCCCCCCCCCCCCCCCCCCTCCCCCCCCCCCCCCCCCC
*N*_44_	T	*N*_47_=T	GGTGGGTGGGGAGGAGGGGGGGGAGGAAAAAAAAAAGAAAGAA
*N*_53_	G	*N*_33_=C	GGGGGGGGGGGGGGGGGGGGGGGGGGGTGGGGGGGGGGGGGG
	T	*N*_33_=T	
*N*_68_	C	*N*_33_=T	GGGGGGGGGGGGGGGGGGGGGGGGGGGCGGGGGGGGGGGGGG
	G	*N*_33_=C	
*N*_146_	G	*N*_38_=A	AAAGGAAAAAAAAAAGAACAAAAAAAAAAAAAAAAAAAAAAAAA

**Fig. 6:**
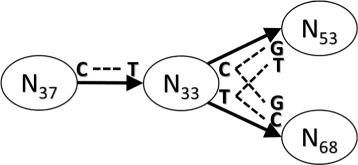
Nucleotides correlation in China data set

**Fig. 7:**
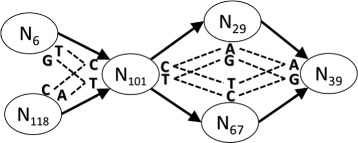
Nucleotides correlation in Korean data set

Finally, the correlation between the nucleotides can be visualized after extracting the prediction rules from the RSGE technique. This form of exploring the effect of nucleotides on one another can provide a better understanding of the mutation mechanism existing in the virus’s RNA. An example of this correlation visual is shown in Fig. 6. In this figure, when the genotype of the nucleotide at position *N*_37_ is C, the genotype of the nucleotide at position *N*_33_ is T. And when the genotype of the nucleotide at position *N*_33_ is T, the genotype of the nucleotides at positions *N*_53_ and *N*_68_ are T and G respectively. The is applied on the rules generated for the Korean data set as shown in Fig. 7.

### NN vs. RSGE

**Fig. 8:**
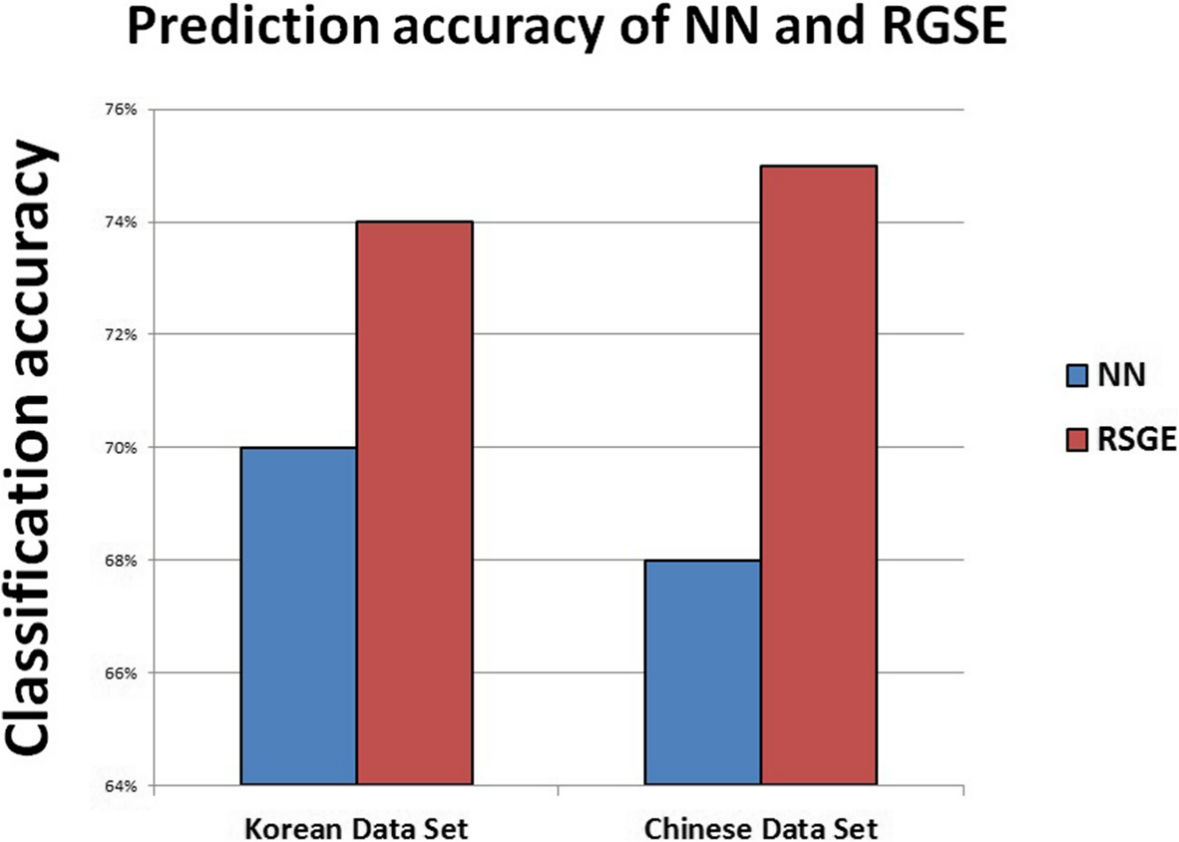
Prediction accuracy for Korean and Chinese data sets

Figure 8 demonstrates a comparison between the results of using neural networks versus the proposed rough set gene evolution prediction techniques in the classification of both the Chinese and Korean data sets. The results show a good performance of the RSGE in comparison to NN. As the data set increase, the preprocessing increases, and hence, the computational complexity of the neural networks and increases the classification accuracy decreases. On the other hand, the error in the classification using the RSGE proposed technique is approximately 2 % in 77 % of the sequence. The rest 23 % could not be predicted by the technique and were replaced by the genotype in the previous generation.

## Conclusions

The contribution of machine learning techniques in the RNA mutation was limited to the prediction of the activity of the virus of resulted RNA. This work paves the way to a new horizon where the prediction of the mutations, such as virus evolution, is possible. It can assist the designing of new drugs for possible drug-resistant strains of the virus before a possible outbreak. Also it can help in devising diagnostics for the early detection of cancer and possibly for the early start-of-treatment. This work studies the correlation between the nucleotides in RNA including the effect of each nucleotide in chaining the genotypes of other nucleotides. These rules of these correlations are explored and visualized for the prediction of the mutations that may appear in the following generations. The prediction rules are extracted by a proposed technique based on RSGE, and is trained by two data sets extracted from two different countries. This work proves the existence of a correlation between the mutation of nucleotides, and successfully predicts the nucleotides in the next generation in the testing parts of two used data sets with a success rate of 75 %. On the other hand, the proposed rough set (RSGE) based technique shows a better prediction result than the neural networks technique, and moreover, it extract the rules used in the prediction.
